# White matter microstructure between the pre‐SMA and the cingulum bundle is related to response conflict in healthy subjects

**DOI:** 10.1002/brb3.375

**Published:** 2015-09-23

**Authors:** Maeri Yamamoto, Itaru Kushima, Hiroki Kimura, Akiko Hayashi, Naoko Kawano, Branko Aleksic, Tetsuya Iidaka, Norio Ozaki

**Affiliations:** ^1^ Department of PsychiatryGraduate School of MedicineNagoya UniversityNagoyaAichiJapan; ^2^ Institute for Advanced ResearchNagoya UniversityNagoyaAichiJapan; ^3^ Institute of Innovation for Future SocietyNagoya UniversityNagoyaAichiJapan

**Keywords:** Attention, connectivity, diffusion tractography, fractional anisotropy, functional MRI

## Abstract

**Introduction:**

Response conflict involves selectively attending to relevant information and suppressing distracting, irrelevant information. The medial frontal cortex (MFC) is considered to be involved in response conflict. However, it remains unclear which white matter connectivity is associated with response conflict. This study aimed to delineate the neural connectivity of response conflict in healthy subjects and investigate the association between white matter microstructure and performance of a response conflict task.

**Method:**

Twenty‐eight healthy subjects underwent functional magnetic resonance imaging (fMRI) during the Flanker task and diffusion MRI. We identified the presupplementary motor area (pre‐SMA) using fMRI. Furthermore, we delineated the white matter connectivity between the pre‐SMA and the cingulum bundle (CB), which is located in the MFC, using probabilistic tractography. We calculated the mean diffusivity (MD), index of white matter microstructure, of this tract and evaluate the association between MD and performance of the Flanker task.

**Result:**

The mean MD of this tract was significantly and positively associated with performance of the Flanker task.

**Conclusion:**

The present study suggests the white matter connectivity between the pre‐SMA and the CB is related to the response conflict in healthy subjects and finer white matter microstructure is associated with smaller response conflict.

## Introduction

Response conflict is the process of selectively attending to relevant information and suppressing distracting, irrelevant information. In everyday life, response conflict is needed in numerous situations, for example, planning, decision‐making, and error detection (Fan et al. 2009). Responses made in the presence of conflict tend to be slower and less accurate (Botvinick et al. 2001). The brain areas involved in response conflict have been investigated, using functional magnetic resonance imaging (fMRI) during tasks such as the Flanker task and the Stroop task. Several fMRI studies have suggested that the medial frontal cortex (MFC) (Ridderinkhof et al. 2004), including the anterior cingulate cortex (ACC) (Casey et al. 2000; van Veen et al. 2001; Bunge et al. 2002; Durston et al. 2003; Weissman et al. 2003) and, more dorsally, the presupplementary motor area (pre‐SMA) (Ullsperger and von Cramon 2001; Garavan et al. 2003; Ridderinkhof et al. 2004; Rushworth et al. 2004; Nachev et al. 2005) are involved in response conflict in healthy subjects. However, it remains unclear which white matter connectivity is associated with response conflict.

Diffusion tensor imaging (DTI) and tractography provide a noninvasive method to track white matter fibers in the human brain and delineate the anatomical connectivity between brain areas (Le Bihan 2003). DTI is based on a model of diffusion in biological tissues and can provide information on white matter microstructure through a variety of parameters (Le Bihan 2003). The parameters most frequently used as indicators of white matter microstructure are mean diffusivity (MD) and fractional anisotropy (FA). MD characterizes overall diffusion and is calculated as the mean of the three eigenvalues of the diffusion tensor. It is considered to reflect white matter density, such as axonal density and myelination (Rae et al. 2015). FA describes the directionality of diffusion and is considered to reflect the degree of myelination, axon diameter, axonal density, and fiber organization (Beaulieu 2002). Lower MD or higher FA implies a finer white matter structure.

Imaging studies have shown that interindividual variation in performance relates to variation in white matter structure in healthy subjects (Johansen‐Berg 2010). Previous DTI studies have suggested a relation between MD and cognitive performance, including post‐error slowing (Fjell et al. 2012), intraindividual variability in reaction time (RT) (Tamnes et al. 2012) and performance of the stop‐signal task (Rae et al. 2015), the cognitive control task (Chaddock‐Heyman et al. 2013), and the Trail Making Test (O'Sullivan et al. 2001), in healthy subjects. In addition, several studies have revealed that FA of a particular white matter region is related to performance of tasks involving motor skills (Doyon et al. 2002; Baird et al. 2005; Schulte et al. 2005; Johansen‐Berg et al. 2007; Della‐Maggiore et al. 2009; Tomassini et al. 2011), memory (Niogi et al. 2008; Fuentemilla et al. 2009; Rudebeck et al. 2009), attention (Madden et al. 2004; Wolbers et al. 2006; Turken et al. 2008; Niogi et al. 2010; Ge et al. 2013), and language or reading (Klingberg et al. 2000; Gold et al. 2007; Floel et al. 2009; Wong et al. 2011).

In this way, that is, by correlation with cognitive performance, DTI parameters can be used to infer the neural substrates underlying specific cognitive functions (Zheng et al. 2014). As such, we can identify white matter pathways related to response conflict by examining the correlation between indices of white matter structure and cognitive performance.

Previous studies using DTI have shown that microstructure in major white matter bundles, such as the anterior corona radiata (ACR) (Niogi et al. 2008, 2010), posterior thalamic radiation, and cerebral peduncle (Chaddock‐Heyman et al. 2013), is related to response conflict in healthy subjects. These DTI studies showed an association between the microstructure of major white matter bundles and response conflict using regions of interest (ROI) defined using a priori knowledge. To the best of our knowledge, there is no study that has shown the relation between response conflict and white matter microstructure of white matter tract that has been delineated, using ROI defined by fMRI in healthy subjects.

The present study aimed to delineate the neural connectivity of response conflict in healthy subjects and investigate the association between white matter microstructure and the performance of a response conflict task. We focused on the major white matter bundle, the cingulum bundle (CB), because it lies in the cingulate cortex, including the ACC, an area that is considered to play an important role in response conflict, and connects the prefrontal, parietal, and temporal cortices, which have been implicated in cognitive control (Schmahmann and Pandya 2006). Indeed, previous DTI studies have shown that the CB is associated with cognitive processes, such as attention and memory, in healthy subjects (Takahashi et al. 2010; Kantarci et al. 2011). We hypothesized that the microstructure of the white matter connectivity between the CB and brain areas in the MFC would be related to the performance of a response conflict task in healthy subjects.

We combined fMRI with an established probabilistic tractography technique (Behrens et al. 2003, 2007) to delineate the white matter connectivity between the CB and brain areas in the MFC. We used fMRI to identify areas of brain activity during the Flanker task in each subject. We selected the Flanker task as a cognitive task for fMRI because it is commonly used to measure response conflict and evokes activation in the MFC. In our study, subjects were required to indicate the direction of a central arrow that was flanked by arrows pointing in the same (congruent) or opposite (incongruent) direction. Response conflict was assessed by subtracting the average RT in the congruent condition from that in the incongruent condition [RT (IC – C)]. A larger difference in the RT indicates more response conflict. We then delineated the white matter connectivity between a region identified as active by fMRI and the CB using DTI in the same subjects. The MD and FA of this white matter tract were computed in each subject and correlated with the degree of response conflict. We predicted that the MD of this white matter tract would positively correlate with RT (IC‐C) and negatively correlate with FA, as white matter connectivity would be related to response conflict and individual differences in white matter microstructure would reflect task performance (Johansen‐Berg 2010).

## Materials and Methods

### Subjects

Twenty‐eight healthy volunteers (16 male and 12 female, mean age 35.7 years, age range 24–48 years, all right‐handed) participated in this study. A psychiatrist confirmed that they had no history of psychiatric or neurological disorders and did not use any psychoactive medications. All subjects provided written informed consent to participate in the study. The Nagoya University Graduate School of Medicine and Nagoya University Hospital ethics review committee approved this study.

### Flanker task

**Figure 1:**
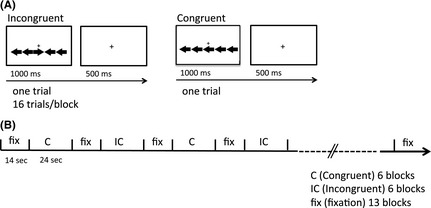
Schematic illustration of incongruent and congruent trials (A) and the experimental paradigm (B).

Subjects underwent a computer‐administered Flanker task in which they were asked to indicate the direction of a centrally presented arrow as quickly and accurately as possible. The stimuli were composed of one target arrow and two distractor arrows on each side of the target arrow (Fig. 1A). On congruent trials, the distractor arrows pointed in the same direction as the target arrow (→→→→→ or ←←←←←). On incongruent trials, the distractor arrows pointed in the opposite direction (←←→←← or →→←→→). On incongruent trials, the distractors caused response conflict and prolonged reaction time. The fMRI experiment was conducted in a block design comprising congruent (C) and incongruent (IC) blocks (block duration, 24 sec). Task blocks were alternated with rest blocks in which fixation was presented for 14 sec (Fig. 1B). In task blocks, the stimulus (target and distractor arrows) appeared randomly above or below the fixation point. For each trial, the stimulus was displayed for 1000 msec and was followed by 500 msec in which only the fixation cross was presented (Fig. 1A). There were 16 trials per block and 12 blocks (six C and six IC blocks) in each run. The experiment consisted of two runs with 192 trials per run. Before the experiment, subjects performed a training phase consisting of 15 trials (eight C and seven IC trials). Accuracy and RT were measured for each trial. Trials with error responses were omitted from analysis. The mean and standard deviation (SD) accuracy is shown in Table 1. Stimulus presentation and response measurement were controlled using Presentation software version 14.4 (Neurobehavioral Systems, San Francisco, CA).

**Table 1:** The results of the Flanker task

	Congruent	Incongruent	Response conflict
Reaction time (ms)	435 (48)	496 (50)^a^	62 (13)
Accuracy (% correct)	99.2 (1.0)	98.1 (3.3)	

Data are mean (SD). Response conflict = reaction time in the incongruent condition – reaction time in the congruent condition.

a Significantly differed from the congruent condition (*P *<* *0.001).

### MRI acquisition

Diffusion MRI and fMRI were acquired in the same session, using a 3‐T MRI scanner (Siemens, Verio, Erlangen, Germany). Diffusion‐weighted data were acquired with the following parameters: b‐value = 1000 s/mm^2^, TR = 9400 ms, TE = 92 ms, FOV = 196 mm, 64 directions, one b0 image, voxel size = 2 × 2 × 2 mm, 60 slices. fMRI data were acquired with the following parameters while the subjects performed the Flanker task: TR = 2500 ms, TE = 30 ms, flip angle 80, FOV = 192 mm, voxel size = 3 × 3 × 3.36 mm, 39 slices, 200 volumes. In addition, a high‐resolution whole‐brain T1‐weighted image was acquired (magnetization‐prepared rapid angle gradient echo sequence: TR = 2500 ms, TE = 2.45 ms, flip angle = 8**°**, matrix = 256 × 256, voxel size = 1 × 1 × 1 mm) for each subject.

### fMRI preprocessing and analysis

Individual fMRI data were processed using SPM8 software (RRID:nif‐0000‐00343; http://www.fil.ion.ucl.ac.uk/spm/software/spm8;%26nbsp;). The first four volumes were discarded to allow for stabilization of the magnetization and successive images in each run were subjected to analysis. First, all the volumes were spatially realigned to the mean volume. The resliced volumes were then normalized to the standard Montreal Neurological Institute (MNI) space, using the transformation matrix obtained from the normalization of the mean EPI image of each subject to the EPI template image. The normalized images were spatially smoothed using an 8‐mm Gaussian kernel.

After preprocessing, statistical analysis of the data obtained for each subject was conducted using a general linear model. At the first level (a fixed‐effects model), each task block was modeled as a hemodynamic response function. High‐pass frequency filters with a cutoff of 128 sec were applied to the time‐series data to remove low‐frequency noise. The images were scaled to a grand mean of 100 overall voxels and scans within a session. In the subsequent analysis, the C and IC conditions were separately modeled as regressors. In addition, the six realignment parameters were used as regressors. The parameter estimates for each condition and for the differences between the conditions were computed from the least‐mean‐square fit of the model to the time‐series data. Images of the parameter estimates representing activity of the task block at each voxel were created for each condition and subject. In the main analysis, we used the difference between IC and C conditions, because the activity indicated by the contrast images reflects the neural correlates of response conflict.

**Figure 2:**
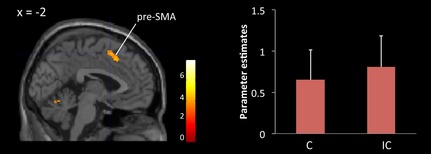
(Left) The pre‐SMA region identified by fMRI group analysis as associated with response conflict shown on the SPM8 canonical template. The statistical threshold was set at *P* = 0.001, uncorrected, and *k* = 30 voxels. (Right) The mean parameter estimate in the pre‐SMA. “C” and “IC” represent congruent and incongruent conditions, respectively. Error bars denote SD.

At the second level of analysis, the contrast images of each subject were entered into a group analysis (a random‐effects model). The contrast images pertaining to the differences in activation between the IC and C conditions were used for the group analysis. A one‐sample t test was performed to test the significance of any difference in activity in the IC and C conditions. Age and gender of subjects were used as covariates. The statistical threshold was set at *P* < 0.001, uncorrected, and *k* = 30 voxels. The results of the subtracted analysis are shown in Table 2. Consistent with previous research (Hazeltine et al. 2000; Ullsperger and von Cramon 2001; Garavan et al. 2003; Ridderinkhof et al. 2004; Rushworth et al. 2004; Nachev et al. 2005), we found robust pre‐SMA activation (*x*,* y*,* z* = −2, 10, 54). A study in the rhesus monkey indicated that the pre‐SMA has connectivity with the cingulate cortex (Morecraft et al. 2012). Therefore, for our subsequent DTI analysis, we selected the peak of the cluster in the pre‐SMA as the ROI associated with response conflict (see Fig. 2, left). Parameter estimates were extracted from the peak voxel in the pre‐SMA, using the MarsBar (radius = 8 mm) toolbox for SPM (http://marsbar.sourceforge.net). The mean and one SD of the parameter estimates for C and IC conditions are plotted in Figure 2 (right).

**Table 2:** The brain regions activated during the Flanker task: Incongruent minus congruent

Region name (Brodmann area)	Hem.	*T*‐value	Coordinates			Size (voxels)
			*x*	*y*	*z*	
Middle occipital gyrus (19)	R	6.84	36	−86	10	3139
Inferior occipital gyrus (17)	L	7.77	−44	−84	−6	1039
Superior parietal lobule (7)	L	5.11	−16	−70	58	669
Medial frontal gyrus (pre‐SMA) (6)	L	4.69	−2	10	54	158
Insula (13)	L	4.20	−34	14	−4	81
Middle frontal gyrus (6)	L	3.74	−30	−2	50	32
Cerebellum	R	4.13	14	−54	−16	113

Hem., hemisphere; L, left; R, right.

*P *=* *0.001 uncorrected, *k *=* *30.

### Diffusion tensor imaging

All DTI data were processed using tools implemented in FSL version 4.1.4 (FMRIB Software Library, RRID:nif‐0000‐00305; http://www.fmrib.ox.ac.uk/fsl). Source data were corrected for eddy currents and head motion by registering all data to the b0 image, with affine transformation. Nonbrain tissues were extracted using the Brain Extraction Tool. The FA maps were calculated using the FMRIB'S diffusion toolbox. The BEDPOSTX tool was applied to estimate diffusion parameters at each voxel (Behrens et al. 2003). The mean FA image of each subject was normalized to the FMRIB58 template in the MNI space using the linear registration algorithm FLIRT (Jenkinson and Smith 2001; Jenkinson et al. 2002).

### ROI mask setting

**Figure 3:**
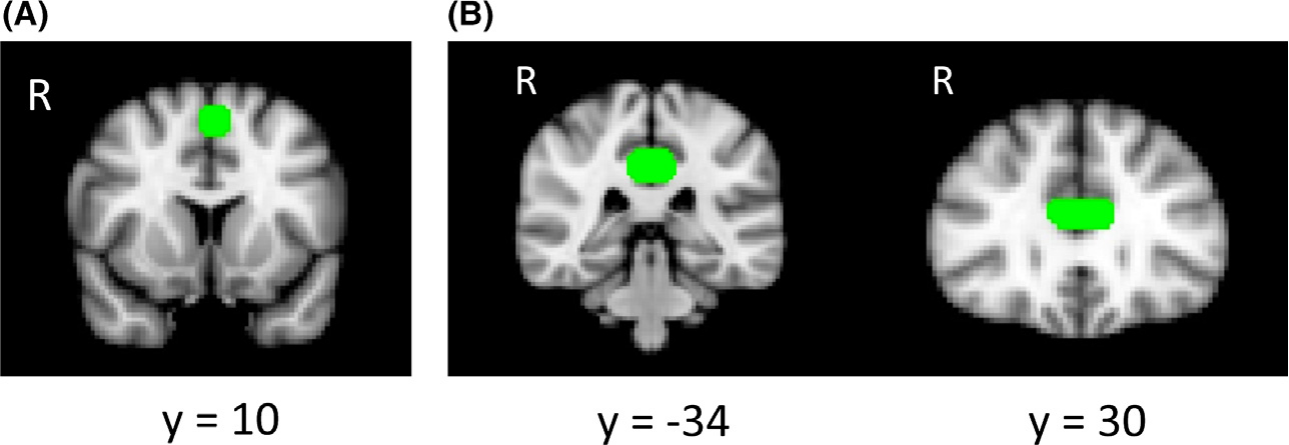
(A) The mask of the pre‐SMA ROI is shown in green on MNI standard space. (spherical *r* = 8 mm). (B) Masks used for the posterior (left) and anterior (right) CB ROI.

We drew three mask images to determine connectivity between the CB and the pre‐SMA: one in the anterior part of the CB (as a seed), one in the posterior part of the CB (as a target) and one in the pre‐SMA (as a waypoint). Masks were drawn by dilating a sphere (radius 8 mm) centered at the pre‐SMA identified from fMRI data using the MarsBar toolbox in MNI space (coordinates, −2, 10, 54). We defined the CB by means of fiber tracking with seed and target ROIs placed over the posterior‐anterior trajectory of the CB. Using FSLView, seed and target masks on the posterior and anterior part of the CB were manually drawn in MNI space based on the Johns Hopkins University tractography atlas, which is provided by FSL. Because the CB atlas of Johns Hopkins University is merged in one single ROI including both hemispheres, we made masks as such. Figure 3 depicts the a priori masks in MNI standard space (A: pre‐SMA, B: anterior [*Y* = 30], and posterior [*Y* = −34] parts of the CB) that were chosen to compute probabilistic trajectories and to generate a network of tracts‐of‐interest for subsequent analyses. Finally, these three MNI space masks were converted to each participant's native space using the inverse matrix of the normalization parameters created during the linear registration process by FMRIB'S linear Image Registration Tool (FLIRT) in FSL.

### Fiber tracking

**Figure 4:**
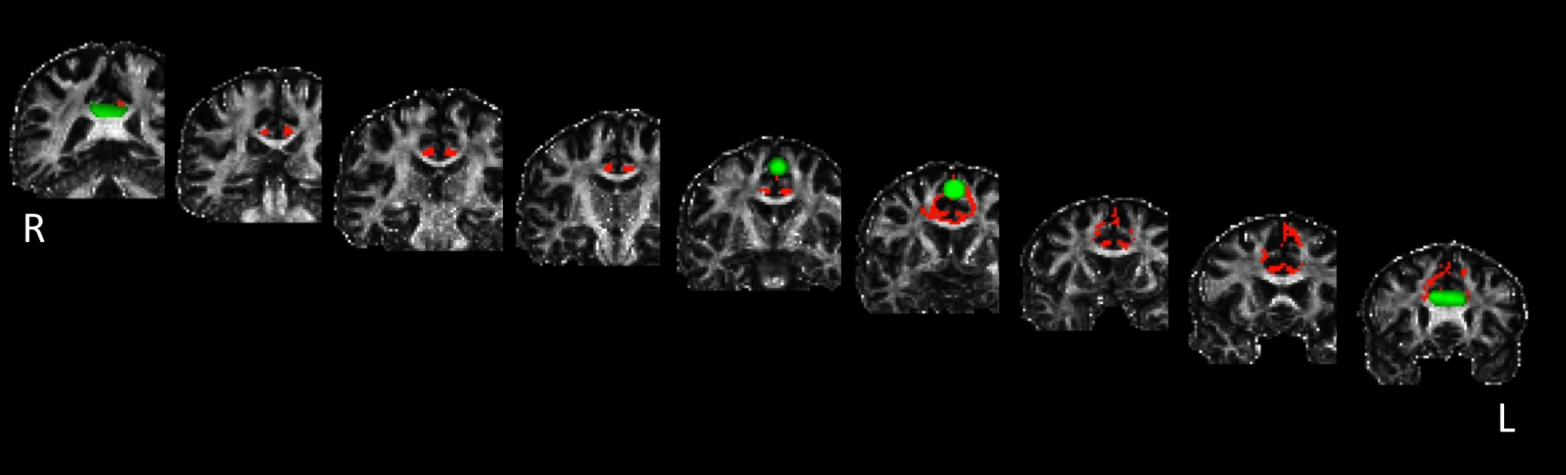
White matter connectivity between the pre‐SMA and the CB in a representative subject is shown in red on the native space. Every third slice (distance = 6 mm) from the posterior to anterior part of the brain is shown. Green areas indicate the masks.

**Figure 5:**
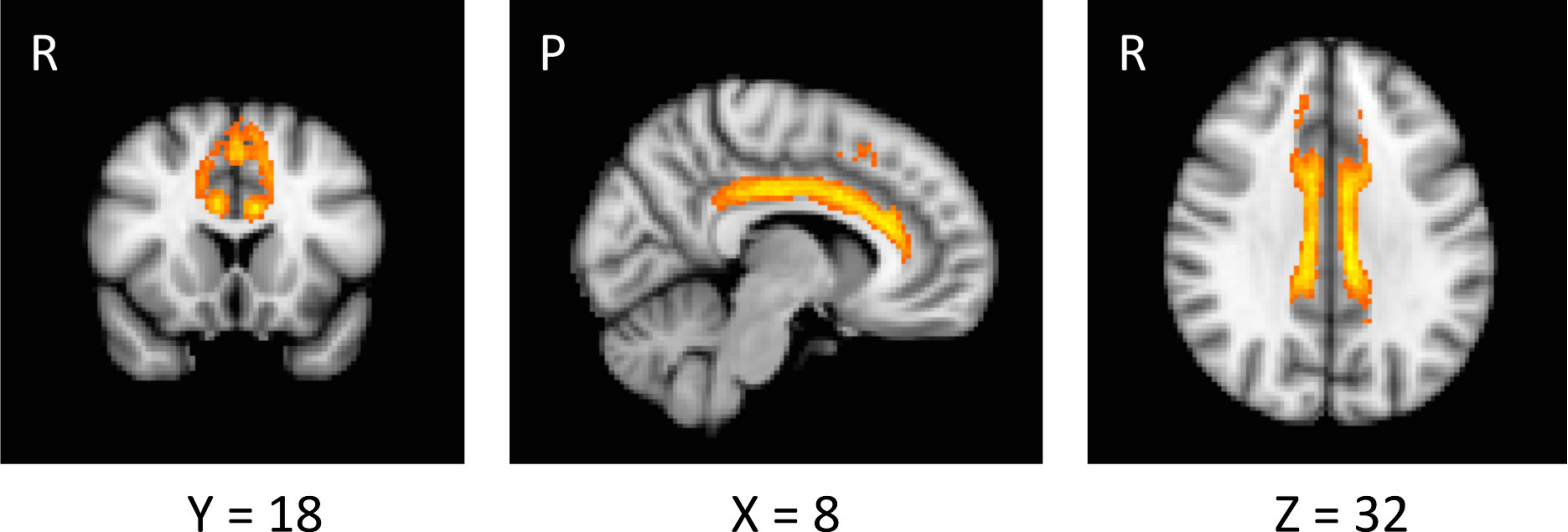
The white matter connectivity between the pre‐SMA and the CB in MNI standard space. The tracts of 64% (18/28) subjects are overlaid on the FSL MNI template.

**Figure 6:**
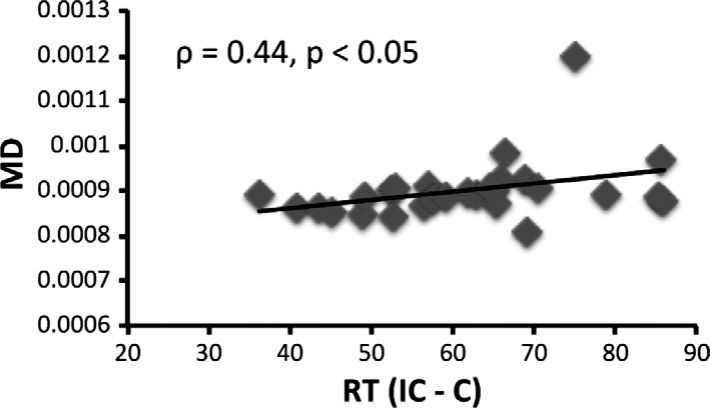
Scatter plot of the mean MD of the pre‐SMA‐CB white matter connectivity and RT (IC – C). The correlation coefficient (Spearman's *ρ*) and statistical significance are shown.

All fiber tracking was conducted in the individual native diffusion space. For each subject, seed and target masks in the CB and the waypoint mask in pre‐SMA were placed on the native space. Fiber tracking was performed using a probabilistic tractography method based on a multifiber model and was applied to the present data using routines implemented in PROBTRACKX (5000 streamline samples, step length = 0.5 mm, curvature threshold = 0.2, maximum number of steps = 2000). The multiple masks option was used to generate a connectivity distribution between seed and target mask images to define the CB, and the waypoint option was used to generate a connectivity passing through the pre‐SMA. This procedure yielded a single cluster of white matter tract in native space for each subject (Fig. 4). For each subject, the cluster of white matter tract was transformed to the MNI standard brain using FLIRT and then binarized. The clusters of white matter tract were then combined into one image in MNI space using fslmaths. In these combined tract images, the intensity at each voxel represents the number of subjects whose tract included this voxel. To remove implausible connections, only voxels that included the tract of at least 18 subjects were retained by fslmaths (Fig. 5). This cluster of white matter tracts was transformed to native diffusion space using FLIRT and binarized. Then, each tract was multiplied by the MD image (L1 image)/FA image, using the fslmaths command in the subject's native space. The mean MD/FA of the tract was computed for each subject, using the fslstats command implemented in FSL. Finally, Spearman's rank correlation coefficient between MD/FA and RT (IC – C) was computed across the 28 subjects (threshold at *P* < 0.05) (Fig. 6).

## Results

### Behavioral results

Table 1 shows the mean (SD) RT for each condition of the Flanker task. A paired *t* test showed that the mean RT was significantly longer for the IC condition than for the C condition (*t* (27) = 24,53, *P* < 0.001). The mean RT (IC – C) was 62 ms. Accuracy did not differ significantly between the conditions (*t* (27) = 2.19, *P* = 0.37).

### fMRI and DTI results

Results from the group analysis of fMRI data are shown in Table 2. Figure 2 (left) shows significant activation associated with response conflict in the pre‐SMA of the left hemisphere. The mean parameter estimate extracted from the peak voxel for the IC and C conditions is shown in Figure 2 (right). DTI analysis revealed the white matter connectivity between the pre‐SMA and anterior and posterior parts of the CB in each subject. The tract in a representative subject is shown in Figure 4. The mean MD of the tract was 0.0009 (SD 0.0007). There was a significant positive correlation between RT (IC – C) and MD (*ρ *= 0.44, *P* < 0.05) (Fig. 6). After excluding an outlier from the analyses, there remained a significant positive correlation between these two variables (*ρ *= 0.41, *P* < 0.05). The mean FA was 0.32 (SD 0.04) and there was no significant correlation between RT (IC – C) and FA (*ρ *= 0.13, *P* = 0.50). There was no significant correlation between RT (IC – C) and the difference in BOLD signal intensity in the pre‐SMA between IC and C conditions (*ρ *= −0.04, *P* = 0.85). The mean MD was not correlated with the subjects’ age (*ρ *= 0.20, *P* = 0.30) and was not different between males and females (*P* = 0.50). RT (IC – C) was not correlated with the subject's age (*ρ * =  0.26, *P* = 0.19).

## Discussion

The present study shows that white matter connectivity between the pre‐SMA and the CB was associated with response conflict in healthy human subjects. The mean MD was significantly and positively correlated with RT (IC – C). Our results show that lower MD, namely, finer white microstructure, was associated with smaller response conflict. These results suggest that this white matter connectivity is related to response conflict in healthy human subjects and that white matter microstructure is related to the performance of a response conflict task.

MD characterizes the overall diffusion and presence of obstacles to diffusion (Le Bihan 2003). In white matter, MD may decrease when axons and glial cells, such as oligodendrocytes, which make up the myelin sheaths, prevent free diffusion. Thus, MD represents the white matter microstructure. MD variation could be related to variation in cognitive performance. Indeed, several DTI studies have suggested a relation between MD and cognitive performance in healthy subjects. Fjell et al. showed that MD in the superior longitudinal fasciculus, inferior fronto‐occipital fasciculus, and anterior thalamic radiation was negatively related to post‐error slowing (Fjell et al. 2012). Rae el al. showed that lower MD in the pre‐SMA and subthalamic nucleus tract, and the inferior frontal gyrus and subthalamic nucleus tract was related to better performance of the stop‐signal task (Rae et al. 2015). Tamnes et al. suggested that lower MD in the forceps minor and the corpus callosum was related to lower intraindividual variability in RT in healthy children and adolescents (Tamnes et al. 2012). In children, smaller MD in the posterior thalamic radiation was associated with greater accuracy in a cognitive control task (Chaddock‐Heyman et al. 2013). MD of anterior white matter positively correlated with executive function assessed by the Trail Making Test in healthy elderly subjects (O'Sullivan et al. 2001). As such, MD in white matter tracts that mediate cognition is associated with cognitive function. Similarly, our results show that lower MD in the white matter connectivity between the pre‐SMA and the CB was related to better performance of a response conflict task.

Contrary to our prediction, there was no significant correlation between FA and the performance of the Flanker task in the present study. We suggest that this unexpected result is because of the diffusion tensor model. A voxel in DTI was 2 × 2 × 2 mm in the present study, and therefore includes thousands of axons, glial cells, and extracellular matrix. One possible problem in the diffusion tensor model is voxels containing crossing fibers (Le Bihan 2003). When a voxel contains crossing fibers, FA may be lowered. Another possible problem is the partial volume effect, which occurs when there is white/gray matter or white matter/cerebrospinal fluid in the same voxel (Alexander et al. 2001) and will also reduce FA. Thus, it is not always the case that low FA indicates disruption of the white matter microstructure, and it is possible that voxels containing crossing fibers and partial volume effects affected FA in our study.

We delineated the white matter connectivity of the pre‐SMA and the CB. A study in rhesus monkey indicated that the pre‐SMA connects mainly with the cingulate proisocortex, cingulate motor areas, supplementary motor area, lateral premotor areas, and the primary motor area. It also connects with the posterior parietal cortex and posterior superior temporal sulcus as well as the supplementary sensory area and insula (Morecraft et al. 2012). In humans, several studies have created diffusion tensor tractography atlases and revealed the pathways of the CB (Catani et al. 2002; Wakana et al. 2004; Catani and Thiebaut de Schotten 2008). These atlases show that the CB contains long fibers connecting the frontal lobe to the temporal lobe and short fibers extending to the medial frontal, parietal, occipital, and temporal cortex, and different parts of the cingulate cortex. These findings support our tractography results, although there are several limitations associated with the diffusion tensor tractography method, as discussed below.

The present study used fMRI to identify the pre‐SMA as a brain area involved in response conflict. We focused on the pre‐SMA for the following reasons. The neurophysiological study of monkeys has suggested a role of the pre‐SMA in resolving conflict (Stuphorn et al. 2000; Isoda and Hikosaka 2007). In human studies, a patient who had a lesion in the right pre‐SMA had impairments in a conflict‐related task (Nachev et al. 2007), and transcranial magnetic stimulation and direct cortical stimulation of the pre‐SMA altered the performance of response conflict tasks (Nachev et al. 2007; Taylor et al. 2007; Usami et al. 2013). Furthermore, fMRI studies have demonstrated activity in the pre‐SMA related to response conflict (Ullsperger and von Cramon 2001; Garavan et al. 2003; Ridderinkhof et al. 2004; Rushworth et al. 2004; Nachev et al. 2005). Thus, recent studies in both species indicate that the pre‐SMA plays a role in resolving response conflict. The present study found robust pre‐SMA activation, but the BOLD signal change in the pre‐SMA between the IC and C conditions did not correlate with response conflict. The relations between BOLD signal intensity and behavioral performance are not consistent across studies in young healthy subjects (Bennett and Rypma 2013). In the present study, the lack of inter‐individual variation in BOLD signal change between conditions may have been due to the simplicity of our task.

Regarding our fMRI result, additional activations were found in the middle/inferior occipital cortex, superior parietal cortex, insula and cerebellum. The aforementioned brain regions seemed to be associated with the performance of the response conflict task. Especially, the superior parietal cortex is considered to be involved in the top‐down visuospatial attention. This region plays a role in orienting attention to the relevant target location in the presence of response conflict (Petersen and Posner 2012). Moreover, the superior parietal cortex forms a frontoparietal network and involved in executive control (Petersen and Posner 2012). Furthermore, the insula is also considered to be involved in this network and engaged in tasks that require controlled processing of information (Cauda et al. 2012). Therefore, there is a possibility these brain regions indirectly influence the performance of the response conflict task. The pre‐SMA along with the ACC has been repeatedly reported to contribute to response conflict (Ullsperger and von Cramon 2001; Garavan et al. 2003; Ridderinkhof et al. 2004; Rushworth et al. 2004; Nachev et al. 2005), so we focused on the pre‐SMA primarily in the present study. We are planning to investigate whether the microstructure of white matter connectivity between these regions may be related to the performance of response conflict task in the next step.

Previous DTI studies have suggested that response conflict is related to microstructure in the ACR in healthy subjects (Niogi et al. 2008, 2010). The ACR is an important projection fiber and runs adjacent to and also synapses in the ACC (Niogi et al. 2008). fMRI studies have reported that the ACC plays a critical role in response conflict, so it is plausible that the ACR is associated with response conflict. We showed that the microstructure of white matter connectivity between the pre‐SMA and the CB was related to response conflict. Considering the function and anatomical connectivity of the pre‐SMA and the CB, white matter connectivity connecting these brain regions would be related to response conflict. Our result is consistent with previous studies in that white matter connectivity connecting the MFC, including the pre‐SMA, and the ACC, is related to response conflict.

The present study has several limitations, mainly associated with the tractography methodology. First, voxels containing crossing fibers may deviate the real course of the fibers. In order to remove spurious streamlines, we adopted a method devised by a previous study (Rae et al. 2015). Second, tractography cannot detect synapses, so it cannot determine the precise termination of connections. This means that it is impossible to determine whether a tract is a continuous long‐range connection or a succession of short fibers (Jbabdi and Johansen‐Berg 2011). Furthermore, tractography is unable to distinguish between efferent and afferent projections. We could not determine whether the white matter tract of the pre‐SMA merged into or passed through the CB. Therefore, there remains a possibility that this connectivity may be a part of the neural network of response conflict. Third, there are several pitfalls involved in combining tractography with fMRI (Catani 2006). We used fMRI activation to determine the waypoint ROI. fMRI is an effective method to detect important information on brain function, but cortical regions have lower anisotropy, which increase the chance of leading to erroneous constructions of tracts. In addition, the use of fMRI to identity active regions was based on group analysis, so it doesn't reflect individual activation. Therefore, further study is required to consider brain activation at an individual level. Finally, there is a limitation associated with setting the ROI. We set ROI masks for the CB based on the Johns Hopkins University tractography atlas. There is a possibility that the tractography of the CB depends on the operator. However, this may be unavoidable because there is no standardized method for mask setting. In diffusion tractography, there are many problems to solve and the results must be interpreted carefully. However tractography is the only tool that allows us to visualize white matter connectivity in‐vivo (Jbabdi and Johansen‐Berg 2011).

In conclusion, our study showed that combined fMRI and DTI can reveal the neuroanatomical substrates of response conflict in healthy subjects. Subjects with small response conflict tended to have finer structural connectivity between the pre‐SMA and the CB compared to subjects with large response conflict.

## Conflict of Interest

None declared.
